# Theoretical design for covering Engeletin with functionalized nanostructure-lipid carriers as neuroprotective agents against Huntington’s disease via the nasal-brain route

**DOI:** 10.3389/fphar.2023.1218625

**Published:** 2023-07-10

**Authors:** Smriti, Madhav Singla, Saurabh Gupta, Omji Porwal, Dalal Nasser Binjawhar, Amany A. Sayed, Pooja Mittal, Fatma M. El-Demerdash, Mohammad Algahtani, Sachin Kumar Singh, Kamal Dua, Gaurav Gupta, Puneet Bawa, Ahmed E. Altyar, Mohamed M. Abdel-Daim

**Affiliations:** ^1^ Chitkara College of Pharmacy, Chitkara University, Rajpura, Punjab, India; ^2^ Chameli Devi Institute of Pharmacy, Department of Pharmacology, Indore, Madhya Pradesh; ^3^ Department of Pharmacognosy, Faculty of Pharmacy, Tishk International University, Erbil, Iraq; ^4^ Department of Chemistry, College of Science, Princess Nourah bint Abdulrahman University, Riyadh, Saudi Arabia; ^5^ Zoology Department, Faculty of Science, Cairo University, Giza, Egypt; ^6^ Department of Environmental Studies, Institute of Graduate Studies and Research, Alexandria University, Alexandria, Egypt; ^7^ Department of Laboratory & Blood Bank, Security Forces Hospital, Mecca, Saudi Arabia; ^8^ School of Pharmaceutical Sciences, Lovely Professional University, Phagwara, Punjab, India; ^9^ Australian Research Consortium in Complementary and Integrative Medicine, Faculty of Health, University of Technology Sydney, Ultimo, NSW, Australia; ^10^ Discipline of Pharmacy, Graduate School of Health, University of Technology Sydney, Ultimo, NSW, Australia; ^11^ Uttaranchal Institute of Pharmaceutical Sciences, Uttaranchal University, Dehradun, India; ^12^ School of Pharmacy, Suresh Gyan Vihar University, Jaipur, India; ^13^ Center for Transdisciplinary Research, Saveetha Dental College, Saveetha Institute of Medical and Technical Sciences, Saveetha University, Chennai, India; ^14^ Center of Excellence for Speech and Multimodel Laboratory, Institute of Engineering and Technology, Chitkara University, Rajpura, Punjab, India; ^15^ Department of Pharmacy Practice, Faculty of Pharmacy, King Abdulaziz University, Jeddah, Saudi Arabia; ^16^ Pharmacy Program, Batterjee Medical College, Jeddah, Saudi Arabia; ^17^ Department of Pharmaceutical Sciences, Pharmacy Program, Batterjee Medical College, Jeddah, Saudi Arabia; ^18^ Pharmacology Department, Faculty of Veterinary Medicine, Suez Canal University, Ismailia, Egypt

**Keywords:** Huntington disease, engeletin, phytoconstituent, neuroprotective, intranasal, nano formulation, nanostructured lipid nanocarrier, formulation

## Abstract

**Objective:** To propose a theoretical formulation of engeletin-nanostructured lipid nanocarriers for improved delivery and increased bioavailability in treating Huntington’s disease (HD).

**Methods:** We conducted a literature review of the pathophysiology of HD and the limitations of currently available medications. We also reviewed the potential therapeutic benefits of engeletin, a flavanol glycoside, in treating HD through the Keap1/nrf2 pathway. We then proposed a theoretical formulation of engeletin-nanostructured lipid nanocarriers for improved delivery across the blood-brain barrier (BBB) and increased bioavailability.

**Results:** HD is an autosomal dominant neurological illness caused by a repetition of the cytosine-adenine-guanine trinucleotide, producing a mutant protein called Huntingtin, which degenerates the brain’s motor and cognitive functions. Excitotoxicity, mitochondrial dysfunction, oxidative stress, elevated concentration of ROS and RNS, neuroinflammation, and protein aggregation significantly impact HD development. Current therapeutic medications can postpone HD symptoms but have long-term adverse effects when used regularly. Herbal medications such as engeletin have drawn attention due to their minimal side effects. Engeletin has been shown to reduce mitochondrial dysfunction and suppress inflammation through the Keap1/NRF2 pathway. However, its limited solubility and permeability hinder it from reaching the target site. A theoretical formulation of engeletin-nanostructured lipid nanocarriers may allow for free transit over the BBB due to offering a similar composition to the natural lipids present in the body a lipid solubility and increase bioavailability, potentially leading to a cure or prevention of HD.

**Conclusion:** The theoretical formulation of engeletin-nanostructured lipid nanocarriers has the potential to improve delivery and increase the bioavailability of engeletin in the treatment of HD, which may lead to a cure or prevention of this fatal illness.

## 1 Introduction

Neurodegenerative diseases (NDG) refer to any irregularities in the brain, spinal column, or other parts of the body that impede the appropriate functioning of the CNS (Thakur et al., 2016). Multiple sclerosis (MS), Parkinson’s disease (PD), Alzheimer’s disease (AD), Huntington’s disease (HD), and brain tumors are believed to be the sources of neurological illnesses (neurogenesis) in the brain, characterized by elevated levels of reactive oxygen species along with neuroinflammation (Sun et al., 2008; Rahman et al., 2022). Neurological conditions are extremely common and disabling, especially in older people (Raggi et al., 2022). According to the WHO, approximately 1 billion individuals worldwide suffer from neurological illnesses, and the frequency of HD varies by geographic region, affecting almost one in every 9 people (Rawlins et al., 2016). According to statistics, Europe and the United States have higher illness rates than China, India, Central Asia, and Africa (Revilla, 2020). The prevalence of 3.92 occurrences per 100,000 people has been estimated globally by compiling all studies since 1985 (Medina et al., 2022). Approximately 40,000–70,000 people in India, or 4–7 out of every 1 lakh, have HD (Rawlins et al., 2016). Huntington’s disease, often known as “Westphal disease,” is an autosomal dominant condition that causes the cytosine-adenine-guanine (CAG) trinucleotide to proliferate, resulting in the creation of a mutant protein known as Huntingtin (Htt), which causes the loss of psychomotor skills (Squitieri et al., 2006). A polyglutamine (polyQ) sequence was obtained from the CAG repeats. In healthy individuals, chromosome 4 of Htt comprises a triplet sequence, and 8–35 CAG trinucleotide repeats can be found in a row. These repetitions are more than 36 in patients with HD due to mutant Huntingtin (mHtt) accumulation (Warby et al., 2009; Reiner et al., 2011). Furthermore, the age at which symptoms manifest and their severity are associated with polyQ repeat expansion. The intensity of HD symptoms and the age at which they appear to increase when polyQ repetitions are longer (Reiner et al., 2011). In the putamen and caudate of the basal ganglia, there is an accumulation of Htt, which leads to the degradation of neurotransmitters, and eventually to the death of neuronal cells. Excitotoxicity may cause neuronal cell death or enhanced signaling in these neurons, resulting in excessive intracellular calcium. Gamma-aminobutyric acid (GABA) and acetylcholine (Ach) levels in afflicted regions have been demonstrated to be lower in patients with HD, but several researchers have observed higher dopamine concentrations (Bains and Shaw, 1997; Landles and Bates, 2004). Adolescent onset accounts for 5%–10% of cases, with symptoms occurring between 10 and 20. Most juvenile instances are transmitted from the father, and patients exhibit bradykinesia, dystopia, tremors, and a cognitive deficiency rather than chorea. Greater hypokinetic symptoms help identify patients with the Westphal variant of HD (Ribaï et al., 2007). Chorea is among the most well-known motor symptoms of HD and is distinguished by uncontrollable muscle spasms that increase with time and interfere with daily tasks (Jankovic and Roos, 2014). Although preventing HD is not attainable, some medications can assist patients in coping with their symptoms (Roos, 2010). Haloperidol, risperidone, rivastigmine, donepezil, amantadine, fluoxetine, buspirone, propranolol, sertraline, tetrabenazine, and clozapine are some of the medications recommended for the treatment of symptoms of HD (Videnovic, 2013). Symptomatic therapies for motor and psychological symptoms may enhance the favourable short-term influence on motor function and quality of life (Bachoud-Levi et al., 2019). However, patients are discouraged from administering the medication due to its widespread adverse effects, which include hallucinations, stomach disturbance, agitation, organ toxicity, drowsiness, dry mouth, tremors, high heart rate, and constipation (Videnovic, 2013). Therapists have been working at the cellular level in previous therapy since the efficiency of Htt-lowering treatments, such as DNA, RNA, or proteins, targets a specific point along the Htt pathway (Wild and Tabrizi, 2017). Recently, there has been a boom in research to improve nutrition and avoid illness. It is commonly acknowledged that plant-based diets play a significant role in preserving health by shielding biological tissues against oxidative stress (OS) and inflammation, two indications of HD (Jang et al., 2021). Lately, polyphenols, particularly resveratrol, have garnered considerable attention due to their neuroprotective effects, particularly in body parts associated with HD’s genesis (Maher et al., 2011). Engeletin (ENG), another isolated compound from grapes (*Vitis*) and wine (*Vitis vinifera*), and later in the leaves of the plant *Engelhardia roxburghiana* (Trousdale and Singleton., 1983; Chamkha et al., 2003; Huang et al., 2011). ENG has curative potential for managing inflammatory and proliferative diseases such as osteoarthritis, endometritis, acute lung injury, cervical carcinogenesis, lung cancer, and AD (Patel, 2022). The ENG induces OS and anti-inflammatory activity by regulating Keap1/Nrf2 pathway (Gupta et al., 2021). Based on current information, ENG may exhibit promising effects against HD irrespective of its limited bioavailability and low aqueous solubility. According to Oshra Betzer’s research, the nano formulation of herbal medications may boost their bioavailability and enable them to penetrate the BBB by lowering their first-pass metabolism (Betzer et al., 2017). Nano emulsions, solid lipid nanoparticles (SLNs), liposomes, and nanostructured lipid nanocarriers (NLCs) are all potential nanostructures for medicinal herb encapsulation (Vishwas et al., 2021). We sought to improve ENG bioavailability using a novel drug delivery technique based on NLC intranasally. In that respect, we reviewed the literature. We identified the cure to the existing problem by theoretically proposing that ENG-NLC, along with some functionalization, would make it potential to deliver to the brain, and the findings were encouraged.

## 2 Pathophysiological changes inside Huntington’s disease

The pathophysiology of HD is specific to the brain and manifests several neuropsychiatric symptoms, including irritability, anxiety, sadness, mental confusion, apathy, and motor dysfunction (Tabrizi et al., 2013; Trovato et al., 2022). The neuronal loss in the striatum and cerebral cortex causes neuronal dysfunction (neurodegeneration) associated with HD (Craufurd et al., 2001). 1993 researchers identified a mutated gene causing HD (MacDonald et al., 1993). The Htt protein has a mass of approximately 350 kDa and is situated in the cytoplasm but has no clear significance. Htt is a pharmacophore protein in several ways because it contains hydrophobic α-helices and numerous HEAT repeats that influence protein-protein interactions (Andrade and Bork, 1995). According to a previous study, HD patients exhibit excitotoxicity, mitochondrial dysfunction, OS, neuroinflammation, genetic factors, protein aggregation, and a lower amount of ATP (Chaturvedi et al., 2009).

### 2.1 Genetic factor’s role in the HD

In 1983, James Gusella’s investigation into the genetic basis of HD indicated for the first time that the Htt gene is connected with a polymorphic DNA marker corresponding to human chromosome 4, precisely 4p16.3 (Gusella et al., 1983). The Huntington’s Disease collaborative Research Group postulated a decade later that the polyQ-coding exon 1 in the N-terminus on the Htt gene is the source of CAG trinucleotide extension that produces triplet repeat family abnormalities (MacDonald et al., 1993). The CAG sequence is repeated 8–34 times in healthy humans, with a median of 16–21 repetitions (Saudou and Humbert, 2016). However, patients often have a CAG expansion ranging from 35 to 120 repetitions. The development of HD is negatively linked to the extension of CAG repeats above the threshold of 35 (Gusella et al., 1983; Rubinsztein et al., 1996; Rosenblatt et al., 2012).

### 2.2 Excitotoxicity role inside the HD

Excessive glutamate receptor activation on postsynaptic membranes is induced by enhanced glutamate production from cortical afferents, decreased glutamate absorption via glia, hypersensitization of voltage-ligand-gated cation channels, and the excitatory neurotransmitter N-methyl-D-aspartate receptor (NMDAR) (Schwarcz et al., 1977; Kalonia et al., 2011; Kumar et al., 2011). The NMDA subclass of ionotropic receptors for glutamate are more sensitive toward voltage-dependent inhibition by extracellular magnesium, highly permeable to calcium ions, and have lethargic activation and deactivation rates (Mayer et al., 1984). The NR1 and NR2 are the components of NMDARs, which form inside the endoplasmic reticulum (ER); they are further classified into 8 splice variants for the NR1 subunit and 4 genes involved for the NR2 subunit (NR2A, B, C, and D) (Hollmann et al., 1993; Monyer et al., 1994). The two most common NR2 subdivisions produced in adult frontal brain tissue are NR2A and NR2B, and the human striatum expresses NR2B more abundantly than the remaining NR2 subunits (Landwehrmeyer et al., 1995; Vincni et al., 1998). Alterations in maintaining calcium concentration in extracellular fluid, depolarization of the mitochondrial membrane, and caspase-3,6 stimulation have all been linked to variations in NMDAR activity (Kumar et al., 2011).

### 2.3 Mitochondrial dysfunctioning role inside the HD

Neurons have important energy requirements, and during oxidative phosphorylation, mitochondria produce adenosine triphosphate (ATP) from metabolic messengers produced in the tricarboxylic acid (TCA) cycle (Mandavilli et al., 2005; Jurcau, 2021). The control of calcium homeostasis also depends on the mitochondria, voltage-gated calcium channels (VGCCs), and liberation of intracellular Ca2+ storage, primarily in the ER, which is involved in calcium influx (Leenders et al., 1986; Nicholls., 2017). The mitochondrial permeability transition (MPT), activated by excessive calcium absorption, destroys the internal mitochondrial membrane electrochemical gradient calcium, and apoptotic agents enter the cytosol (Duchen, 2004; Orrenius, 2004). They even noticed impaired glucose breakdown and reduced mitochondrial oxygen consumption within the brains of HD patients’ (Stahl and Swanson, 1974; Tabrizi et al., 1999). Moreover, measurements of energy metabolism parameters revealed decreased cyclic adenosine monophosphate (cAMP) levels in cerebrospinal fluid (CSF) (Cramer et al., 1984). HD brain’s striatum and cerebral cortex exhibit increased polyQ levels (Nicholls., 2017). The efficiency of oxidative phosphorylation-related enzymes, such as aconitase and mitochondrial complexes II, III, and IV, has been reported to be reduced in post-mortem striatal tissue from patients of HD (Brennan et al., 1985; Tabrizi et al., 1999; Akhlaq et al., 2021). MHtt genes act in cells at the cellular level, and this protein interacts with the mitochondrial transporter II receptor causing oxidative damage and mitochondrial malfunction in HD patients (Tabrizi et al., 1999; Nicholls, 2017).

### 2.4 Oxidative stress role inside the HD

However, the functionality of the OS in HD remains unclear. According to certain molecular theories, superoxide anion radicals are generated when oxygen sometimes absorbs electrons in the electron transport chain (ETC). Manganese superoxide (O2^–^) dismutase converts these radicals into hydrogen peroxide in the mitochondria. Glutathione peroxidase or peroxiredoxins act in the mitochondria transforming hydrogen peroxide to water (Santos et al., 2003; Mandavilli et al., 2005). ETC protein inhibition can exacerbate dopamine metabolism, lipids’ peroxidation, and ROS production (DiFiglia, 1990; Kumar et al., 2010). Similarly, increased amounts of 3-nitrotyrosine, a marker of RNS, are detected in the cortex and striatum (Browne et al., 1997). The cell also features non-enzymatic ROS scavengers, including GSH, ubiquinone, vitamin E, and C. Therefore, OS arises from a disparity between the formation of ROS and antioxidant activity (Ott et al., 2007; Scherz-Shouval and Elazar., 2007). mHtt protein accumulates in HD patients, which may also contribute to an increase in OS (Browne et al., 1999; Chen, 2011). Free radical concentration increases, leading to energy deficits and metabolic inhibition (Sawa et al., 2003; Túnez et al., 2010).

### 2.5 Neuroinflammation role inside the HD

Post-mortem examinations have revealed a high concentration of active immune cells in the decaying HD neurons, particularly microglia and macrophages. The brain’s macrophages, known as microglia, activate in Htt (Li et al., 2011; Chen et al., 2014; Jamwal and Kumar, 2015). Increased microglia counts and morphological alterations often indicate microglia activation in tissue specimens (Bjorkqvist et al., 2008). The dysregulation of cytokine production in the striatum and cortex of patients suffering from HD includes IL-6 (interleukin-6), IL-1, IL-8, MMP-9, and TNF α (Kalonia et al., 2011a; Kalonia et al., 2011b; Mehta et al., 2013; Chen et al., 2014). Neurons and astrocytes control microglia’s effects via cytokine mediators and neurotransmitters, which cause intricate interactions among microglia, neurons, and astrocytes. This delicate relationship is called neuroinflammation (Carson, 2002; Garden, 2002).

### 2.6 Protein aggregation role inside the HD

**FIGURE 1:**
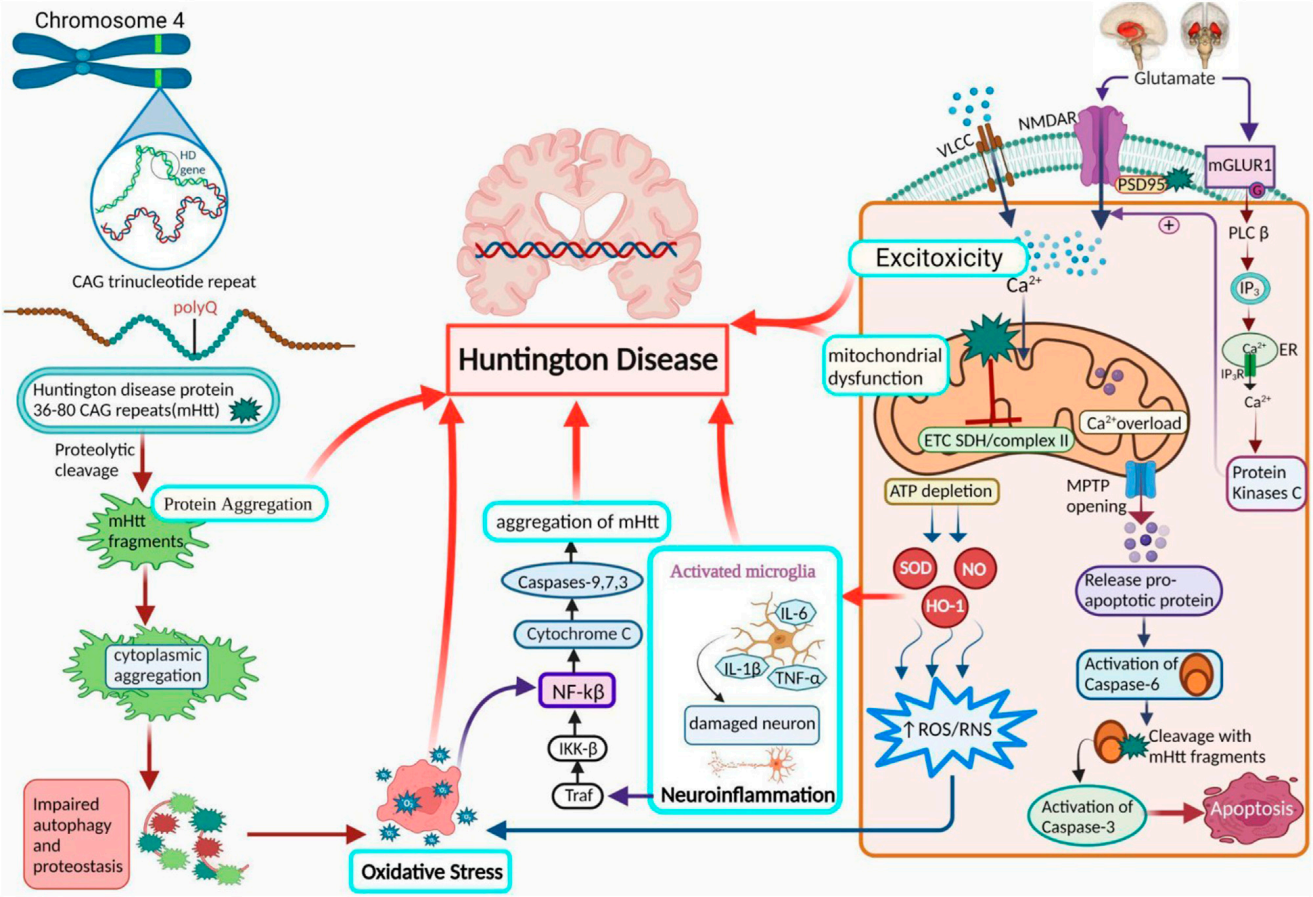
Pathogenesis of HD. A diagram demonstrates the role of activated microglial cells, neuroinflammation, OS, protein aggregation, excitotoxicity, and mitochondrial dysfunction in HD. PolyQ repetitions leading to the accumulation of Htt (protein aggregation) are the core reason behind the progression of HD. Ca2+ homeostasis is abnormally influenced by Htt due to an increase in Ca2+ influx by NMDA receptors, resulting in excitotoxicity. It also results in mitochondrial dysfunction via a variety of mechanisms. Further HD leading factors include suppressed ETC and complex II, along with a rise in neuroinflammation and oxidative stress. NMDAR: *N*-methyl-D-aspartate receptor, NF-κB: nuclear factor kappa-light-chain-enhancer of activated B cells; NO: nitric oxide; ROS: reactive oxygen species; Cx-II: succinate-ubiquinone oxidoreductase.

Inherent aspects that influence mHtt aggregation include polyQ expansion, surrounded by the amino acid sequestrate and altered configuration of mHtt (Scherzinger et al., 1999; Bhattacharyya et al., 2006; Rockabrand et al., 2007). Longer repetitions manifest early illness, more severe pathophysiology, and rapid neurodegeneration (Rosenblatt et al., 2012). Among these, the expansion of polyQ plays a significant role. When the polyQ extension exceeded a certain length, a switch occurred from the potential random coil structure to the β-sheet structure. Misfolded protein-protein interactions occur, resulting in protein aggregation, which is caused by the altered protein structure; it is initiated by proteolytic cleavage and several other proteases, including caspases, calpains, and aspartic proteases (Perutz et al., 1994; Martindale et al., 1998; Wellington et al., 2002.; Gafni et al., 2004; Nagai et al., 2007) (Figure 1).

## 3 Engeletin: A miraculous polyphenol

**FIGURE 2:**
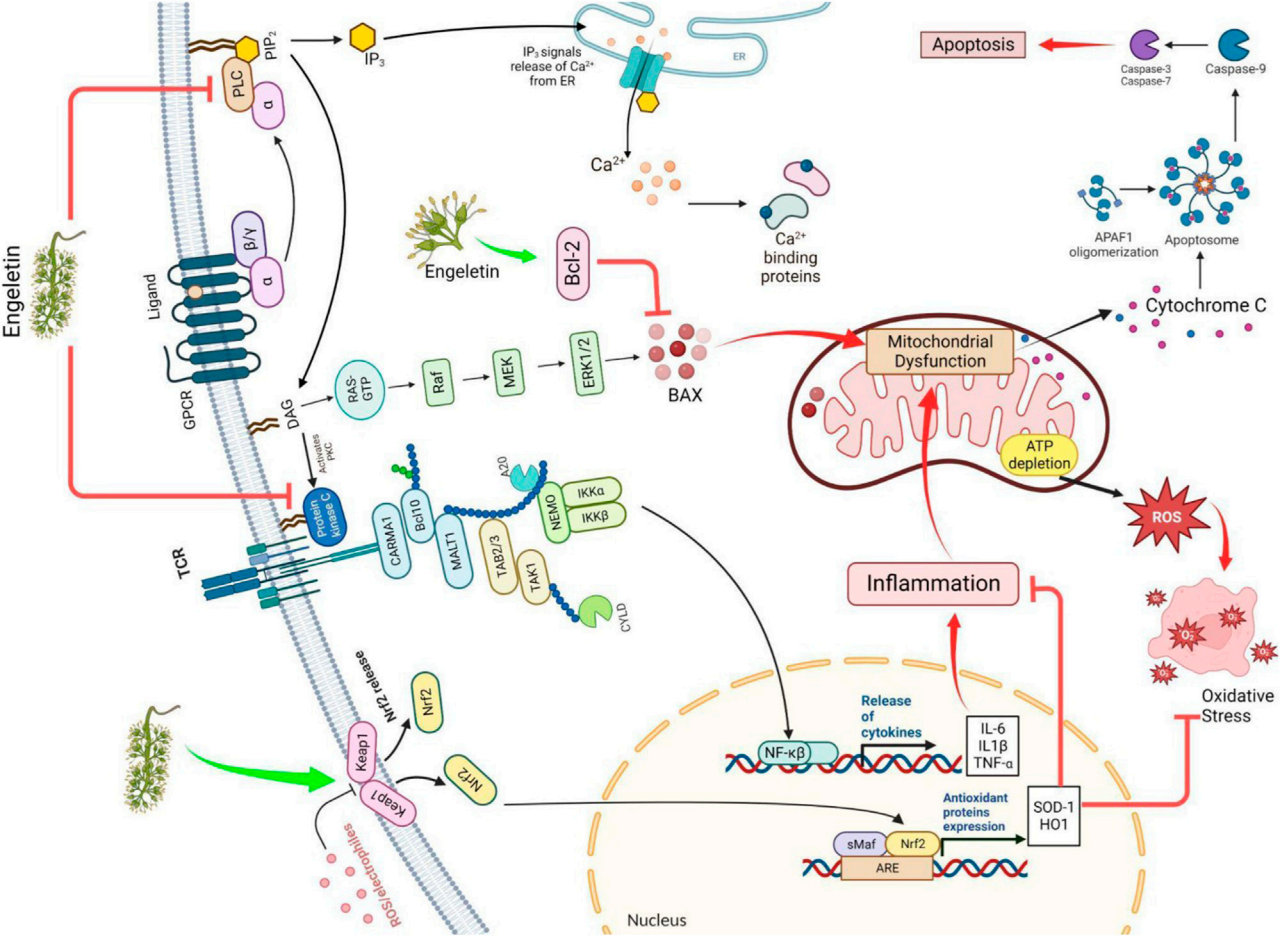
The figure illustrates how ENG improves HD by modulating the Nrf/keap and Bcl2 pathways and blocking the IKK, PLC, and PKC pathways. ROS: reactive oxygen species; Nrf/Keap: nuclear factor erythroid 2–related factor/Kelch-like ECH-associated protein; ARE: antioxidant response element; IKK: IκB kinase; RAS: renin-angiotensin system; Raf: rapidly accelerated fibrosarcoma, sMaf: small musculoaponeurotic fibrosarcoma; MEK: mitogen-activated protein kinase kinase; ERK: extracellular signal-regulated kinase; IL: interleukin; PKC: protein kinase C; PLC: phospholipase C; Bcl2: B-cell leukemia/lymphoma 2 protein; BAX: Bcl-2-associated X protein.

A flavanol glycoside/polyphenol, ENG (3-0-a-1-Rhamnosyl-(2R, 3R)-dihydro kaempferol), may be extracted from the bark of Hymenaea martiana if the appropriate conditions are met (Carneiro et al., 1993). It has been shown that it may be present in white *Vitis* (grapes) as well as in white *Vitis vinifera* (wine) skin (Eugene and Trousdale Vernon, 1983). The leaves are used to make a sweet tea called Huang qi in China, which aids weight management, and a tea called Kohki in Japan. It is widely accessible across Southeast Asia (Huang et al., 2011). It has been proven that ENG exhibits multiple biological actions, including prevention against inflammation and analgesic, diuretic, and antibiotic properties. Moreover, it contains a wide range of biological capabilities, including properties acting against oxidation and cancer, and it can safeguard the liver and lungs from damage (Cox et al., 2005; Li et al., 2007; Peng et al., 2012; Jiang et al., 2018; Tian et al., 2019; Bai and Yin, 2020.; Liu et al., 2020). ENG, a naturally occurring mitochondrial antioxidant, may penetrate the membrane and accumulate in mitochondria *in vitro* or *in vivo*. This study examined whether the ENG target may reduce inflammation induction by inhibiting the free radical formation and downregulating cytokine (inflammatory mediators)(Patel., 2022). This hypothesis states that ENG inhibits mitochondrial dysfunction and reduces calcium loading capacity by suppressing inflammatory mediators. ENG suppresses neuroinflammatory responses in glial cells by inhibiting Phospholipase C (PLC) and Nuclear factor kappa B (NF-kB) via the Keap-Nrf pathway (Zhu et al., 2010; Wang et al., 2020). Glial cells are stimulated when Htt protein is present. This enhances antigen activation and inflammatory mediators’ release (Banati, 2002). These secretions interact with T-cells, neural progenitor cells, glial cells, and astrocytes in the surrounding region. These mediators may cause extensive brain damage due to chronic inflammation, cell death, free radical formation, NMDA-mediated excitotoxicity, and caspase stimulation. This study investigated whether ENG activation reduces glial cell activity and functions as a potential medicinal molecule for treating patients suffering from HD. Aldose reductase(AR) is pivotal in the beginning and progression of inflammation caused by OS via NF-κB-dependent expression of inflammatory mediators (Yadav et al., 2009). ENG, a naturally occurring AR inhibitor, suppresses NF-kB-dependent inflammatory signals caused via cytokines, growth regulators, and endotoxins and possesses anti-inflammatory activity (Ramana and Srivastava, 2010). A completely distinct pathway was discovered to be effective in neuroprotection, and releases ARE, including SOD-1 and HO-1, via the Keap pathway (Zhu et al., 2010). A hypothesized portrayal of the function of ENG in HD treatment is depicted in Figure 2.

## 4 Theoretical ground justification

### 4.1 Improving mitochondrial functioning

Numerous histological and morphological analyses of cortical samples from patients with HD have revealed aberrant mitochondrial morphology. The Caudate and putamen nuclei of post-mortem HD brains disclose complexes II and III activity, significantly decreased due to oxidative phosphorylation. Succinate dehydrogenase is a key factor that downregulates the ETC and tricarboxylic acid (TCA) cycle enzymes (Olesen et al., 2022). Considering excitotoxicity, Novelli et al. discovered in 1988 that factors impacting energy metabolism, including instability of oxidative metabolism and the Na+/K+ ATPase pump in cells, may result in excitotoxicity (Novelli et al., 1988; Huang et al., 2011). Mitochondrial dysfunction and excitotoxicity are major contributors to HD pathogenesis.

HD cell and animal models have convincingly demonstrated that excitotoxicity and mitochondrial dysfunction occur early in the disease cycle and are believed to have a connection to the pathology of the illness. HD’s hyperkinetic and hypokinetic symptoms can develop or be spawned by 3-Nitropropionic Acid (NP), a non-reversible blocker of succinate dehydrogenase (Borlongan et al., 1977; Ludolph et al., 1991). A small dose of 3-Nitropropionic Acid administered chronically to rats and non-human primates induces lasting metabolic changes and other features comparable to people suffering from HD (Beal, 1994; Brouillet et al., 1998). Moreover, a rise in calcium concentration opens voltage-gated NMDA channel complexes, causing excitotoxicity and its accompanying processes, such as stimulating proteases like calpain. In turn, calpains facilitate the breakdown of many proteins, including Htt (Túnez et al., 2010).

Further production of Htt impairs mitochondrial dysfunction, which leads to oxidative and nitrative stress, thereby modifying oxidative metabolism. Inhibiting the enzyme ETC causes the formation of several electrons, oxygen, and hydrogen peroxide ions, resulting in an imbalance between antioxidants and oxidants. Carbonylated proteins, lipid peroxides, and 8-hydroxy-2-deoxyguanosine (8OHdG) have been identified as contributing to oxidative damage (Coles et al., 1979; Halliwell., 2006; Moncada and Bolanos, 2006). All these alterations result in the creation of ROS and RNS, which cause neural cell death. These findings reinforce the hypothesis that the synthesis of mHtt affects cell energy rates and may even cause excitotoxic death as it impairs calcium processing and mitochondrial energy metabolism. Here, ENG acts as an antioxidant and anti-apoptotic agent, neutralizing reactive species (Li et al., 2022). Hence, ENG is a therapeutic agent and prevents neuronal death in HD patients.

### 4.2 Suppression of inflammation via Keap1/nrf2 pathway

Glial cells are vital for developing and efficiently performing neural mechanisms, and their contributions cannot be overstated. However, often in neurodegenerative disorders, the modulation of these cells is disrupted, contributing to brain pathology (Wake et al., 2011). HD is distinguished by the development of neuropathic phenotypes in glial cells, resulting in the discontinuation of the regulatory roles of these cells. Therefore, glial cells have a significant function in the aetiology of HD because of the production of mHtt in these cells. These cells create the same aspects of the aetiology of the disease, including motor and cognitive impairment (Savage et al., 2020). Glial cell activation occurs through the amplification of cell surface antigens and the generation of inflammatory mediators. These cytokines include TNF-α, IL-6, chemokines, inducible nitric oxide synthase (iNOS), and cyclooxygenase-2 (COX-2) (Subhramanyam et al., 2019). The NF-κB pathway is active in both neural and glial cells (O'Neill and Kaltschmidt, 1997). Neurite expansion and neuronal survival depend on NF-κB pathway activity (Teng and Tang, 2010). Modification in NF-κB signaling has been linked with the loss of striatal neurons in HD (Yu et al., 2000). Ali Khoshnan and his colleagues demonstrated that the NF-κB pathway is crucial to this neuroprotective response. It has been established that mHtt produced from an inducible promoter could stimulate NF-κB in a brain cell line.

Furthermore, empirical evidence has indicated that blocking the NF-κB pathway may lower mHtt toxicity (Khoshnan et al., 2004). Intracellular signaling through these pathways is a major contributor to inflammation because it induces NF-κB, a critical regulation of cytokine production. Signal transmission occurs through the adaptor proteins MyD88 and IRAK1, which phosphorylate and activate IKK in response to Toll-like receptor (TLR) activation. Free NF-κB molecules may initiate gene transcription in the nucleus (Hayden and Ghosh, 2012). Ulrike Trager and his colleagues demonstrated that mHtt binds IKKγ, which increases the rate of IKKβ degradation and hence boosts NF-κB activity. Immune cells release more cytokines because of the altered transcription of NF-κB target genes. In HD, transcriptional dysregulation induced by the NF-κB pathway upregulates seven genes (TLR2, LTBR, CD40, TMED4, AKT1, IL10, and FR2), but CHUK is significantly downregulated (Träger et al., 2014). After the release of pro-inflammatory mediators, there is a possibility that inflammation, free radicals production, the excitotoxicity driven by NMDA, and the activation of caspase take place and may, all of which may lead to degeneration of neurons and damage to tissue throughout the brain (Kim and de Vellis, 2005; Zhu et al., 2010). Through the Keap1-Nrf2 pathway, ENG suppresses the synthesis of inflammatory mediators, such as TNF-α, IL-1β, and IL-6. Superoxide dismutase (SOD) and Glutathione peroxidase (GSH-Px) are two antioxidant enzymes generated after the Keap1-Nrf2 pathway is activated. These enzymes effectively regulate the cytokines responsible for inflammation, which help safeguard cells from free radical damage and inhibit ROS-causing OS (Huang et al., 2020) (Table 1).

**TABLE 1:** Engeletin’s *in vivo* and *in vitro* preventive properties against several disorders.

S.no	Condition	Application (*in vitro*/*in vivo*)	Biological sex	Findings	References
1	Pelvic inflammatory disease	*In vivo*	Female Sprague-Dawley rats	Constraining the PLC/PKC pathway, reducing nuclear translocation of NF-κB, p65, thus revoking the rise in p38, ERK, and JNK phosphorylation	Wang et al. (2020)
		*In vitro*	RAW 264.7 murine macrophages	Reduced generation of inflammatory mediators and improved phagocytic capability	
2	Pulmonary fibrosis	*In vivo*	C57BL/6 mice	By lowering amounts of fibrotic markers like α-SMA, vimentin, and collagen as well as raising levels of E-cadherin in epithelial cells, ENG eventually prevents fibrogenesis	Zhang et al. (2020)
		*In vitro*	L929 mouse fibroblast cell line	It stifled the growth and movement of stimulated fibroblasts, hence preventing fibrogenesis	
3	Arrhythmia	*In vivo*	Male C57BL/6 mice	By improving cardiac structure, electrical and ion channel reconfiguration, and OS, ENG reduced the vulnerability to ventricular fibrillation	Fang et al. (2023)
		*In vitro*	H9C2 cells	ENG increases the capability to get rid of ROS, boost the efficiency of ARE and minimizes the levels of MDA and GSSG via suppressing the Nrf2 signal - ML385	
4	Mucin gene	*In vitro*	NCI-H292 cells	ENG suppresses human airway mucin gene expression via regulating hydrolysis of IkBα and NF-kB	Hossain et al. (2022)
5	Cerebral ischemia	*In vivo*	Male Sprague-Dawley rats	By promoting angiogenesis through VEGF/vasohibin signaling, ENG therapy controlled cerebral flow of the blood, neural function, and life span stability	Liu et al. (2021)
		*In vitro*	Human umbilical vein endothelial cell	The angiogenesis-regulating processes of HUVEC migration, proliferation, and tube formation were all facilitated through the VEGF/vasohibin as well as Ang-1/Tie-2 pathways	
6	Osteoarthritis	*In vivo*	Male Sprague-Dawley rats	ENG may decrease the progression of osteoarthritis induced by an anterior cruciate ligament (ACLT) injury	Wang et al. (2021)
		*In vitro*	chondrocytes	Extracellular matrix disintegration, apoptosis, mitochondrial membrane potential degradation, and ROS production all decreased following TNF-α exposure in chondrocytes treated with ENG. Cartilage was preserved by stimulating Nrf2 and decreasing the NF-kB and MAPK pathways	
7	Ferroptosis in bone marrow mesenchymal stem cells	*In vivo*	Sprague-Dawley rats	The expression of TFR1 was found to be reduced, while that of FPN1, GPX4, and SLC7A11 all upregulated, suggesting that ENG may effectively prevent OS damage to cells. It boosts the lifespan of mesenchymal stem cells derived through bone marrow by reducing their rate of ferroptosis	Huang et al. (2023)
8	Endometritis	*In vivo*	Female BALB/c mouse	ENG significantly decreases uterine pathology, has anti-inflammatory characteristics, modulates inflammatory cytokines that are released, and may work by reducing NF-κB	Wu et al. (2016)
9	Alzheimer disease	*In vitro*	BV-2 cell line	OS was suppressed by lowering ROS and MDA production while enhancing GSH-Px and SOD activity. Reduced generation of TNF-, IL-1, and IL-6, together with decreased NO fabrication and iNOS expression, indicate that it also dampens the inflammatory response	Zhu et al. (2010)
10	Liver injury	*In vivo*	Male BALB/c mice	The anti-inflammatory activities of ENG were driven by stimulation of PPAR-γ, which reduced inflammatory mediator production and serum ALT and AST levels	Tian et al. (2019)
11	diabetes[α-glucosidase inhibitors]	*In vitro (molecular docking)*	AUTODOCK 4.2 software	ENG suppresses α-glucosidase noncompetitively via creating bonds of hydrogen and hydrophobic interactions with certain amino acid residues	Li et al. (2021)

## 5 Theoretical and experimental challenges with engeletin

Although ENG is a medicinal plant with various therapeutic characteristics, it has some challenges associated with its physicochemical properties, such as limited bioavailability, low aqueous solubility, need for BBB permeability, and poor pharmacokinetics (Ye et al., 2017; Ye et al., 2019; Shen et al., 2021). Due to its presence at minimal levels in brain tissue, ENG is currently being researched as a possible therapy for neurological illnesses, despite these shortcomings (Ye et al., 2019). Innovative drug delivery techniques for herbal encapsulation have been significantly advanced. Regarding bioavailability, toxicity, pharmacological activity, prolonged administration, and physical and chemical degradation, novel formulations exceed standard and antecedent methods (Rahman et al., 2020). It was initially expected that nanocarriers would be the most convenient option to conquer the problems and constraints of present drug delivery systems, enhance medicine bioavailability and therapeutic efficacy, and provide preferential accumulation at the target site (Ni, 2017). Multiple studies have shown that the pharmacokinetic characteristics of phytoconstituents can be modified to increase their oral bioavailability. Since various polyphenols, flavanols, and glycosides have been used to treat other neurodegenerative illnesses, they are expected to effectively treat HD via novel drug delivery systems (NDDS) (Debnath et al., 2017). Research on delivery methods, including ENG, is essential to treat HD effectively. Preclinical studies are the only source of information regarding nanoparticle formulations for HD treatment.

## 6 Need of NDDS (novel drug delivery system) for the formulation of engeletin

Various medicinal herbs provide many therapeutic benefits to the human body, with fewer side effects than synthetic ones. Phytoconstituents obtained from plant sources are currently in demand for treating various elementary disorders. The phytoconstituents have previously been acquired in small amounts. Some possess great *in vitro* bioactivity but little or no *in vivo* activity due to their limited bioavailability and poor physicochemical characteristics, including solubility, permeability, and disintegration (Kumar et al., 2019; Srivastava et al., 2019). Extensive research is now being conducted in the area of novel delivery of drugs for targeting plant extracts and actives. It is the most appropriate method to convey the primary component of a herbal medication on the desired site of action at a rate regulated by the body’s needs. Nanonization of herbal medicines has become a powerful tool for developing new drug delivery systems and has attracted considerable interest recently. It can overcome the major limiting variables (lipid solubility and molecular size) for therapeutic molecules to penetrate the cellular membrane and be systemically absorbed after oral or topical administration. It has several other benefits, such as boosting chemical solubility, decreasing medicinal uses, and enhancing the absorption of herbal medicines compared to the corresponding crude medication formulations (Watroly et al., 2021). Hence, herbal preparations may be more effective in the human body if the phytoconstituent drugs are delivered via NDDS.

## 7 Nanocarrier technology-based delivery systems for the treatment of neurological disorders

Millions worldwide suffer from bothersome and crippling illnesses, including neurodegenerative disorders. Numerous studies have shown that various nanoformulations made from naturally occurring polyphenols, including curcumin, quercetin, and resveratrol, dramatically improve the health of individuals with neurological diseases. Medicinal herbs can be encapsulated or loaded in nanostructures, including nanoemulsions, SLNs, liposomes, and NLCs (Dewi et al., 2022). Bioavailability is the principal limitation influencing the translational perspectives of these herbs, with the BBB being the primary obstruction. The BBB comprises a monolayer of brain capillary endothelial cells with few pores and extensive tight junctions encircled by blood capillaries. This prevents bulkier or hydrophilic molecules from engaging with the BBB, which, as a result, penetrates it (Brookes et al., 2022). Therefore, drugs with high lipophilicity, low molecular weight, hydrogen bonds, and smaller dimensions can traverse the BBB better.

Many nano formulations have been investigated and listed as the most popular way to facilitate drug delivery more precisely and consistently to desired body parts, especially the brain. NLCs technology is the most appropriate formulation for our purposes because of its high lipophilicity. NLCs comprise a combination of solid and liquid lipids mixed with a water phase containing a surfactant to create an unstructured solid-lipid matrix (Beloqui et al., 2016; Haider et al., 2020). Lipids are the chief constituent of NLCs, which control the drug loading capacity, duration of action, and formulation stability (Noor et al., 2017). The lipid type and structure regulate various nanocarrier qualities. Generally considered safe (GRAS), biodegradable, non-toxic, and physiologically acceptable lipids have been suggested to produce lipid nanoparticles (Shah et al., 2015). During NLC synthesis, liquid lipids are incorporated to form an amorphous structure in the solid core matrix.

In contrast to the pure crystalline solid matrix of SLNs, which has a limited spatial capacity, this allows for higher drug loading while retaining the nanocarrier’s physical stability (Beloqui et al., 2016; Haider et al., 2020). NLCs may be formed from a range of solid lipids such as triglycerides (tristearin), fatty acids (stearic acid), waxes (carnauba wax), and steroids (cholesterol). Liquid lipids include medium-chain triglycerides (such as miglyol 812), oils from natural sources (such as olive oil), fatty acids (such as oleic acids), and other oily substances (such as paraffin oil (Khosa et al., 2018; Haider et al., 2020). NLCs are affordable technologies that can reduce insolubility and enhance the biological availability of drugs and phytoconstituents. It can deliver drugs to their target site with maximum efficiency, which results in improved therapeutic efficacy and avoids the ejection of the therapeutic molecule during storage. It also leads to sustained release, making it well-suited for short-lived drugs.

They exhibit greater permeability across cell membranes, allowing for more efficient drug delivery. Moreover, they can be tailored to fit the needs of different drugs, allowing them to be used in various applications. Finally, using NLCs eliminates the need for toxic surfactants and other additives, making them safe and effective delivery systems (Chauhan et al., 2020).

The unique composition of NLCs allows them to be investigated for their potential to revolutionize the delivery of medicines through several routes of administration. These techniques include topical, nasal, inhalation, and oral administration (Gomaa et al., 2022). Systemic drug delivery via the nasal route is conceivable because the nasal mucosa is rather porous with ample blood perfusion, allowing quick medication uptake into the blood circulation. A potential option, the nasal route, allows medications to be directly administered into the brain, circumventing the BBB and avoiding metabolism, making it appealing. Extensive research has been conducted on the potential advantages of administering drugs through the nasal route for targeted delivery to the brain (Cunha et al., 2021). A nasal spray is the preferred inhalational route because it provides sustained release of drugs and protects molecules from enzymatic decomposition owing to its protective shell. It enhances drug bioavailability by extending the time for the medication to remain in the nasal passage (Cunha et al., 2020). Nasal spraying has been proved innumerous research to be an efficient method of medication delivery to the brain for various neurodegenerative conditions (Mishra et al., 2019; Palagati et al., 2019).

Therapeutics are chemically coupled to antibodies or ligands to target a particular receptor and endocytose (Oberoi et al., 2015). Transferrin receptors, low-density lipoprotein (LDL) receptors, insulin receptors, and lactoferrin receptors are a few of the receptors on brain endothelial cells that may be used for receptor-mediated transcytosis all over the BBB (Niu et al., 2019). Moreover, receptor-mediated transcytosis initiates the entry of various proteins, including transferrin, insulin, leptin, and lipoproteins, via the vesicular trafficking systems of the brain. Harmonizing ligands with their natural receptors on the brain can significantly improve the supply of therapeutics to the brain, particularly macromolecular therapies (Lajoie and Shusta, 2015; Zhu et al., 2019). ENG-encapsulated NLCs to the brain because of their advantageous properties, particularly the avoidance of lysosomal degradation along the route of low-density lipoprotein (LDL) delivery to the brain, which permits the sustained release of carrying ligands to brain targets (André et al., 2020). apoB and apoE are among the ligands recognized by the transmembrane receptor LDLR (Go and Mani, 2012). Due to the NLC attaching to them, caveolae-mediated endocytosis of these particles occurs. Targeting LDLR is more successful than targeting other receptors because high blood levels of Tf may inhibit synthetic ligands from connecting to the TfR, even though targeting IR has detrimental side effects such as hypoglycemia (Carr et al., 2000; Go and Mani, 2012; André et al., 2020).

## 8 Theoretical modified proposed formulation of engeletin against HD

Since ENG is both a polyphenol and a flavonol glycoside, it is reasonable to hypothesize that it may be encapsulated in NLCs and delivered via intranasal administration to the brain as a nasal spray, as outlined above. Our objective is to provide a better formulation against HD that patients can afford and self-administer in the case of long-term treatment. Liquid lipid is the core component of the NLCs, according to the literature finding Vitamin E to be the most suitable for our purpose, as it exhibits some exclusive properties, including its highly efficient antioxidant property, which delays the neuronal damage caused by OS. It also enhances the immunomodulatory effects of NCs by increasing the chemical stability of entrapped drugs (Dewi et al., 2022). Since vitamin E dissolves in lipids, it can be detected in LDL. It promises to minimize the responsiveness of LDL to OS, commonly known as lipid peroxidation, which occurs because of the formation of free radicals by macrophages and endothelial cells in the arterial wall (Cunha et al., 2021). According to the existing data, the LDLR pathway may exhibit a significant role in the cellular absorption of α-tocopherol (an isomer of Vitamin E) from LDL (Palagati et al., 2019; Cunha et al., 2020). Thus, NLCs made of vitamin E may show higher selectivity towards LDL receptors. Researchers have investigated high liquid lipid concentrations and determined they have a high therapeutic retention rate because drugs often had better solubility in liquid lipids than solid lipids. NLCs become less viscous and have less surface tension, which makes the particle size smaller (Dewi et al., 2022).

Furthermore, surfactants play a role in drug toxicity, physical stability, dissolution, and permeability profiles, making them a crucial component of NLCs. To stabilize the nanoparticles, the quality of the interfaces must be improved (Chauhan et al., 2020). Tween 80 met our requirements regarding polarity, molecular mass, and suitability for the mode of administration in our composition. As a non-ionic surfactant, it is a hydrophobic tail that stabilizes the particles and prevents them from adhering together. It has been proven beneficial in several formulations. The co-surfactant Phospholipon^®^ 90G was used in the formulation owing to its exclusive ability to emulsify the chosen lipid mixture.

Additionally, it overcomes one of the drawbacks of intranasal administration by not irritating the nasal mucosa. Sara Cunha et al. could design a formulation using the material mentioned above, except NLCs, that carry polyphenols to treat neurological disorders (Cunha et al., 2020). We intended to design a formulation similar to deliver ENG to the brain through the nasal pathway.

**FIGURE 3:**
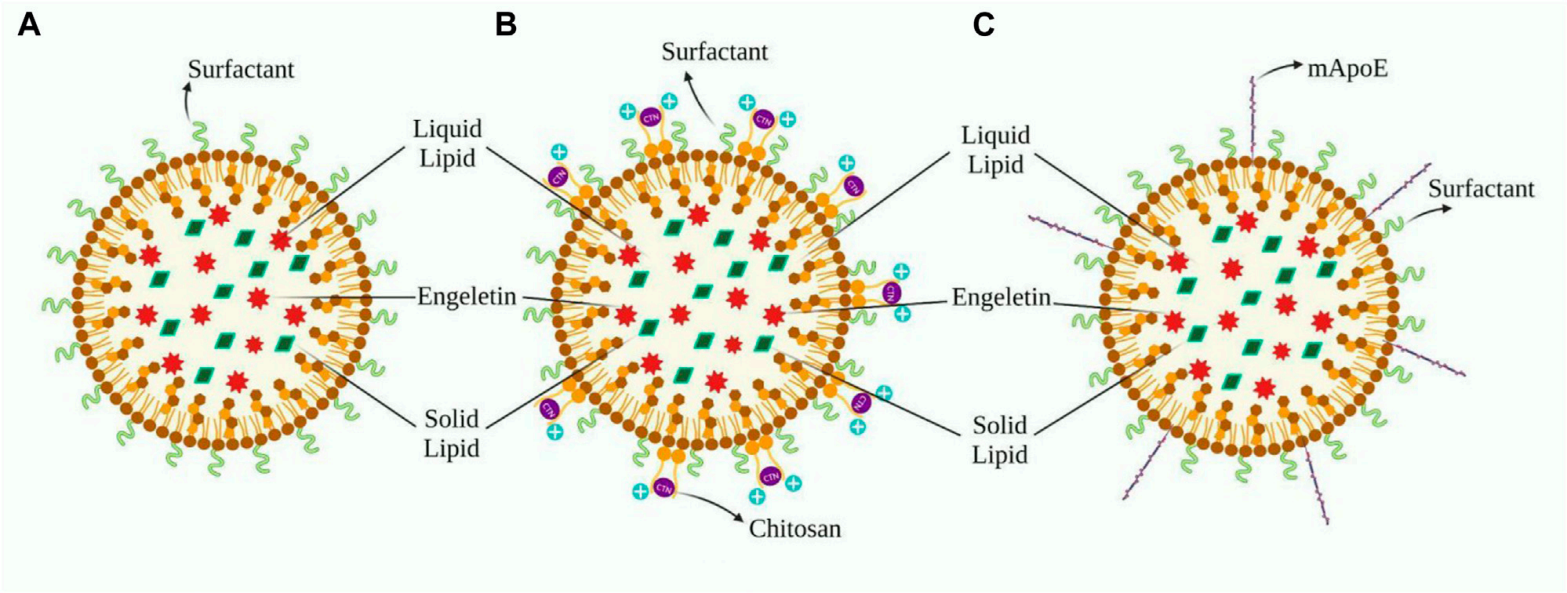
**(A)** Design of ENG’s nanoformulation based on Sara Cunha’s work, whereas B and C are modifications aimed to facilitate distribution across the BBB. **(B)** This depicts the application of a chitosan layer to the outer surface of **(A)** in order to create a cationic nanoformulation. **(C)**) Demonstrates the fictionalization of **(A)** with the inclusion of mApoE, which improves the formulation’s affinity for LDL.

It is possible to modify the outer surface of NLCs so that they can flow more easily through the BBB. Because endothelial cells in cerebral capillaries have a negative charge, adsorption-mediated transport may promote NLC uptake in the brain (Traber and Kayden, 1984; Thellman and Shireman, 1985; Shuvaev et al., 2015). A tiny cation particle with a positive charge must be coated to benefit from it. Chitosan (CTN) is an excellent option for our needs and is often seen in novel formulations. Gartziandia’s research resulted in a CTN-coated NLCs formulation that penetrated intranasally to the brain, whereas Elnaggar’s research encapsulated piperine in monodisperse CTN nanoparticles for intranasal administration to the brain (Elnagger et al., 2015; Gartziandia et al., 2015) Cognitive performance was boosted in healthy rats following the nasal route, which was more effective than standard pharmaceutical therapy (Elnagger et al., 2015). Lipid formulations with a surface charge modulation, particularly cationic elements, successfully overcome complexities, including mucociliary clearance and a short residence timespan in the nasal cavity after administration, thus ensuring maximum drug absorption. Furthermore, nasal toxicity and hemolytic testing revealed that the formulation was safe for intranasal use (Gartziandia et al., 2015). Beatrice Formicola and colleagues suggested another modification of NLC that surface functionalization by aligning with mApoE boosts nanosystem endocytosis by increasing BBB model permeability through LDLR as LDL has high affinity towards ApoE (Formicola et al., 2019) (Figure 3).

## 9 Conclusion

Huntington’s disease (HD), an incurable and progressive neurodegenerative disorder, lacks medication capable of completely halting its development. *In vivo* and *in vitro* research has revealed that certain plants with potent antioxidant and neuroprotective properties may mitigate HD symptoms positively. One naturally occurring molecule called ENG, known for its neuroprotective properties, shows potential in preventing disease progression by targeting disease-causing pathways. Despite its limited solubility in water and low bioavailability, ENG can still exert its protective effects through various pathways. To efficiently deliver the active component of herbal treatment to the specific affected area at a rate tailored to the body’s requirements, nanoscale drug delivery systems (NDDS) are the most effective method. Therefore, we propose a theoretical formulation of ENG that enables uninterrupted transmission across the blood-brain barrier (BBB). However, extensive preclinical and clinical evaluations of nanostructures are necessary to assess potential risks and harmful effects associated with their use. Nevertheless, the proposed framework offers a promising avenue for the future development of nanonized ENG, empowering physicians to overcome current therapeutic obstacles in treating HD.

## Figures and Tables

**Table:** 

**AD**	Alzheimer disease
**Ach**	Acetylcholine
**ApoE**	Apolipoprotein E
**ALT**	Alanine transaminase
**AST**	Aspartate aminotransferase
**AR**	Aldose reductase
**BBB**	Blood brain barrier
**CAG**	Cytosine adenine guanine
**CTN**	Chitosan
**CAMP**	Cyclic adenosine monophosphate
**CNS**	Central nervous system
**CSF**	Cerebrospinal fluid
**COX-2**	Cyclooxygenase-2
**DNA**	Deoxyribonucleic acid
**ENG**	Engeletin
**ER**	Endoplasmic reticulum
**ERK**	Extracellular signal-regulated kinase
**ETC**	Electron transport chain
**GABA**	Gamma-aminobutyric acid
**GSH-Px**	Glutathione peroxidase
**GSSG**	Glutathione disulfide
**HD**	Huntington disease
**Htt**	Huntingtin
**HUVEC**	Human umbilical vein endothelial cells
**IL**	Interleukin
**iNOS**	Inducible nitric oxide synthase
**IKK**	IκB kinase
**LDL**	Low-density lipoprotein
**MDA**	Malondialdehyde
**mHtt**	Mutant huntingtin
**MMP**	Matrix metalloproteinases
**MS**	Multiple sclerosis
**mPT**	Mitochondrial permeability transition
**MAPK**	Mitogen-activated protein kinases
**NLC**	Nanostructured lipid nanocarriers
**NDG**	Neurodegenerative disease
**NDDS**	Novel drug delivery system
**nrf2**	Nuclear factor erythroid 2-related factor 2
**NF-κB**	Nuclear factor kappa-light-chain-enhancer of activated B cells
**NO**	Nitric oxide
**NMDAR**	N-methyl-D-aspartate receptor
**NF-kB**	Nuclear factor kappa B
**OS**	Oxidative stress
**8OHdG**	8-hydroxy-2-deoxyguanosine
**PD**	Parkinson disease
**PolyQ**	Polyglutamine
**PLC**	Phospholipase C
**PPAR**	Peroxisome proliferator-activated receptors
**ROS**	Reactive oxygen species
**RNS**	Reactive nitrative species
**RNA**	Ribonucleic acid
**SOD**	Superoxide dismutase
**SLN**	Solid lipid nanoparticles
**TNF α**	Tumor necrosis factor-alpha
**TCA**	Tricarboxylic cycle
**TLR**	Toll-like receptor
**VGCC**	Voltage-gated calcium channels
**VEGF**	Vascular endothelial growth factor

## Data Availability

The original contributions presented in the study are included in the article/supplementary material, further inquiries can be directed to the corresponding authors.
